# Semaphorin-5A maintains epithelial phenotype of malignant pancreatic cancer cells

**DOI:** 10.1186/s12885-018-5204-x

**Published:** 2018-12-22

**Authors:** Sugandha Saxena, Abhilasha Purohit, Michelle L. Varney, Yuri Hayashi, Rakesh K. Singh

**Affiliations:** 1 Department of Pathology and Microbiology, University of Nebraska Medical Center, Omaha, NE 68198-5845 USA; 2 The Wistar Institute of Anatomy and Biology, Philadelphia, PA 19104 USA; 3 Department of Internal Medicine, University of Nebraska Medical Center, Omaha, NE 68198-5845 USA

**Keywords:** Pancreatic cancer, Semaphorin-5A, Tumor growth and metastasis, Epithelial-to-mesenchymal transition (EMT)

## Abstract

**Background:**

Pancreatic cancer (PC) is a highly aggressive disease, and the lethality of this disease stems from early metastatic dissemination where surgical removal cannot provide a cure. Improvement of the therapeutic outcome and overall survival of PC patients requires to understand the fundamental processes that lead to metastasis such as the gain of cellular migration ability. One such family of proteins, which are essential players of cellular migration, is Semaphorin. Previously, we have identified one of the Semaphorin family member, Semaphorin-5A (SEMA5A) to be involved in organ-specific homing during PC metastasis. We have also demonstrated that SEMA5A has a constitutive expression in PC cell lines derived from metastatic sites in comparison with low endogenous expression in the primary tumor-derived cell line. In this study, we examined whether constitutive SEMA5A expression in metastatic PC cells regulates tumor growth and metastatic potential.

**Methods:**

We generated SEMA5A knockdown in T3M-4 and CD18/HPAF cells and assessed their phenotypes on in vitro motility, tumor growth, and metastatic progression.

**Results:**

In contrary to our initial expectations, orthotopic injection of SEMA5A knockdown cells into nude mice resulted in a significant increase in both tumor burden and liver metastases in comparison with the Control cells. Similarly, we observed higher in vitro migratory potential with pronounced morphological changes associated with epithelial-mesenchymal transition (EMT), a decrease in the expression of epithelial marker E-cadherin (E-Cad), increase in the expression of mesenchymal markers N-cadherin (N-Cad) and Snail and the activation of the Wnt-signaling pathway in SEMA5A knockdown cells. Furthermore, re-establishing SEMA5A expression with a knockdown resistant mouse Sema5A in SEMA5A knockdown cells resulted in a reversion to the epithelial state (mesenchymal-epithelial transition; MET), as indicated by the rescue of E-Cad expression and a decrease in N-Cad and Snail expression.

**Conclusions:**

Collectively, our data suggest that SEMA5A expression maintains epithelial phenotype in the metastatic microenvironment.

**Electronic supplementary material:**

The online version of this article (10.1186/s12885-018-5204-x) contains supplementary material, which is available to authorized users.

## Background

Pancreatic Cancer (PC) is an invariably lethal disease [1] with a five-year survival rate of 7% [2]. These low survival rates are attributed in part of the fact that PC metastases have often progressed to the point where surgical removal cannot provide a cure [1]. Furthermore, exploration and understanding of the molecules and mechanisms that lead to metastatic dissemination in PC are necessary.

A growing body of evidence now supports the role of semaphorins, axon guidance cue molecules, in metastasis [3]. SEMA5A is a novel member of this family and studies on various cancer types have suggested both tumor suppressive and oncogenic roles for SEMA5A [4]. Previous reports from our laboratory have shown that ectopic overexpression of both membrane-bound and secretory form of Sema5A in PC cell line enhances metastasis [5, 6]. We also observed that the secretory form of Sema5A enhances proliferation of endothelial cells and upregulates secretion of angiogenic factors like CXCL8 and VEGF in cancer cells [7]. However, the functional significance of SEMA5A in PC expressing higher endogenous levels of SEMA5A remained unexplored.

In the present study, we tested the hypothesis that knockdown of endogenous SEMA5A expression can modulate tumor growth and metastasis in PC. Towards this end, we performed in vitro and in vivo studies to understand the role of SEMA5A on PC metastasis by generating SEMA5A knockdown cells in PC cell lines. In contrary to our expectation, loss of SEMA5A enhanced metastatic potential of the PC cell by mediating epithelial to mesenchymal transition (EMT). Thus, context-dependent expression of SEMA5A molecule regulates metastatic potential of PC cells by switching cells between epithelial and mesenchymal phenotypes.

## Methods

### Cell culture

The Pancreatic Ductal Adenocarcinoma cell lines T3M-4 and CD18/HPAF cells were a generous obtained from Dr. Hollinsworth and Dr. Batra’s laboratory respectively, University of Nebraska Medical Center; UNMC, Omaha, NE, USA. Use of these cell lines did not require any additional ethics approval. We maintained these cell lines as an adherent monolayer in Dulbecco’s Modified Eagle Medium (Hyclone Laboratories, Logan, UT). The medium was supplemented with 5% fetal bovine serum (Sigma, St. Louis, MO), 1% of 100X MEM vitamin solution (Mediatech, Herdon, VA), 1% of 200 mM L-glutamine and 0.02% gentamycin (Invitrogen, Carlsbad, CA). We tested the cell lines for mycoplasma using MycoAlert Plus Mycoplasma Detection kit (Lonza). For cell line authentication, Human DNA Identification Laboratory, University of Nebraska Medical Center, Omaha, NE, USA performed the short tandem repeat (STR) tests. They evaluated fifteen STR markers (D8S1179, D21S11, D7S820, CSF1PO, D3S1358, THO1, D13S317, D16S539, D2S1338, D19S433, vWA, TPOX, D18S51, D5S818, FGA) and the gender marker Amelogenin.

### Cloning of SEMA5A shRNA oligos

SEMA5A specific (5’-GCG GAT TTC TCG CAG TTA A-3′) oligonucleotide was selected using OligoEngine software (OligoEngine Inc., Seattle, WA) and inserted downstream of the start codon in the pSUPER.neo vector, as instructed by the manufacturer. The oligonucleotide was annealed and cloned into the *Bgl*II and *Hin*dIII restriction site using enzyme digested pSUPER.neo vector. We used vector pSUPER.neo without insert as a negative control for SEMA5A knockdown and verified the sequences.

### Transfection of PC cells

We transfected T3M-4 and CD18/HPAF cells with pSUPER.neo-shSEMA5A or control pSUPER.neo vector using LipofectAMINE Plus reagent (Invitrogen, Carlsbad, CA) according to the protocol provided by the manufacturer. Stably transfected cells were selected and maintained in 1000 μg/mL and 600 μg/mL neomycin sulfate (G418, Mediatech) containing media for T3M-4 cells and CD18/HPAF cells respectively. Similarly, shSEMA5A and Control (empty vector) T3M-4 cells were transiently transfected with FLAG-tagged cDNA containing full-length mouse Sema5A in pBK-CMV vector (a generous gift from Dr. Andreas W. Püschel, Westfälische Wilhelms-Universität Münster, Germany).

### mRNA analysis

Total cellular RNA was isolated from cells using TRIzol® reagent (Invitrogen, Carlsbad, CA). Cells were washed three times with Phosphate Buffer Saline (PBS) made in DEPC-treated water. Cells were scraped using one mL of Trizol and RNA isolated using manufacturer’s instruction. RNA was resuspended in 20 μL DEPC containing water, quantified, and checked for the quality of the RNA obtained. The cDNA was synthesized using two μg of the obtained RNA for a 20 μL reaction using iScript™ Reverse Transcription Supermix for RT-qPCR (BIO-RAD, Hercules, CA, USA). qRT-PCR reactions were prepared using FastStart SYBR Green Master Mix (Roche; Indianapolis IN, USA) and performed using the BIO-RAD Connect machine. BIO-RAD RFX Manager 3.1 software was used to run the analysis. We have listed the details of primer sets used for the study in Additional file 1. For real-time PCR, mean C_t_ values of the target genes were normalized to mean C_t_ values of the endogenous control, Hypoxanthine-guanine phosphoribosyltransferase(HPRT); [−∆C_t_ = C_t_ (HPRT) – C_t_ (target gene)]. We calculated the ratio of mRNA expression of target genes versus HPRT (2^(−∆Ct)^). Melting curve analysis was performed to check the specificity of the amplified product. We resolved the amplified cDNA on EtBr containing 1% agarose gels.

### Western blot analysis

We carried out Western Blot analysis as described previously [7]. Membranes were incubated with following primary antibodies for overnight at 4 °C: SEMA5A (Invitrogen; 1:1000), anti-E-cadherin (E-cad) (HECD1; 1:100), anti-N-cadherin (N-cad) (1:100), anti-β-catenin (1:200), anti-SNAIL (Abcam; 1:500), anti-GAPDH (Santa Cruz, 1:1000) and anti-β-actin (Sigma, 1;1000). PI3K (Sigma, 1:1000), p-PI3K (p85Tyr458-p55Tyr199, Cell Signaling, 1;750), AKT (Cell Signaling 1:1000), p-AKT (Ser473, Cell Signaling, 1:1000), GSK3β (Cell Signaling, 1:1000) p-GSK3β (Ser9, Cell Signaling 1:1000). Anti-E-cad, anti-N-cad, and anti-β-catenin were generous gift from Dr. Keith Johnson, University of Nebraska Medical Center; UNMC, Omaha, NE, USA. The membrane was incubated with secondary horseradish peroxidase antibody (mouse (Sigma), 1:5000, rabbit (Thermo Scientific); 1:5000) for an hour at room temperature. We utilized NIH Image J Software for quantification of the intensity of the bands, of our protein of interest and their respective loading control. We also normalized the bands to the Control cells used in the study.

### Immunofluorescence analysis

We plated cells (5 × 10^5^) on 22 × 22 mm sized coverslips (Fisherbrand ®, Pittsburgh, PA) placed in 6-well plates and the protocol is described previously in detail [8]. We used primary antibodies like, 1:25; E-cad, 1:5; N-cad, 1:5; β-catenin, 1:10; Fascin (Developmental Studies Hybridoma Bank) 1:20; and phalloidin (Texas Red, Molecular Probes) 1:50, and Secondary antibodies conjugated with either mouse or rabbit Cy3 (Jackson Immuno Research). Immunofluorescence localization was determined using either a Nikon Eclipse E800 Fluorescent microscope or LSM 710 Zeiss Confocal Microscope.

We used the same number of cells as described above for quantification of Lamellipodium and Filopodium; actin cytoskeleton was stained using Texas Red Phalloidin for 20 min. We quantified the number of peripheral cells in a colony, Lamellipodia, and Filopodia with a free edge in 5 different random fields in the same coverslip at 630× resolution using LSM 710 Zeiss Confocal Microscope for T3M-4-Control and T3M-4-shSEMA5A cells. Similarly, for CD18/HPAF-Control and SEMA5A knockdown cells, we have used Nikon Eclipse E800 fluorescent microscope at 400× resolution and counted different colonies with a number of cells at periphery and lamellipodia in seven different random fields. We determined the average number of Lamellipodia per cell.

### Cell motility assays

We analyzed cellular motility using Wound Healing assay and Transwell Migration assay. For Wound Healing assay, cells (0.2 × 10^6^ for T3M-4-cells and 0.5 × 10^6^ for CD18/HPAF cells) were plated per well in 12-well plate. The following day, the cells had reached 90–95% confluency. A wound was generated using a one mL pipette tip. Cells were washed with HBSS and incubated with a fresh serum-free medium for 24 h. Cells were photographed under an inverted TS100 microscope (Nikon, Melville, NY) at a 40× magnification at time *T* = 0 h and *T* = 24 h. The distance was evaluated at time 0 h (T_0_) and 24 h (T_24_) using Image J software (NIH, Bethesda, MD). The percent of distance migrated was calculated using the formula: 100 x[(T_0_)–(T_24_)]/ (T_0_).

For Transwell Migration assay, we plated 0.2 × 10^6^ cells/well in the top chamber of noncoated polyethylene terephthalate membranes (six-well insert, 8 μm pore size; Becton Dickinson, Franklin Lakes, NJ) with the bottom chamber containing 1.0 ml of serum-free media. The cells were incubated overnight at 37 °C. After removal of non-migrated cells, membrane pores were stained using Hema 3 kit (Fisher Scientific Company L.L.C., Kalamazoo, MI) as per the manufacturer’s instructions. Cells were counted in ten random fields (100x) and expressed as the average number of cells per field of view. Representative data is the average of independent experiments.

### Cell viability assay

We seeded cells in 96-well plates at low density (2000–4000 cells/well). Following overnight adherence, we incubated the cells with media alone or medium containing different serum concentrations (0–5%) for 24 or 48 h. We determined cell viability using MTT ((3-(4,5-Dimethylthiazol-2-yl)-2,5-Diphenyltetrazolium Bromide, EMD Millipore Corp, Burlington, MA) reagent as described previously in [9]. Data presented as absorbance at 570 nm.

### Propidium iodide staining

Seeded 1X10^6^ cells in a 6-well plate for overnight. Following morning, cells were harvested, washed in PBS and fixed in cold 70% ethanol. Cells were added drop-wise to the 70% ethanol solution while vortexing to ensure homogenous fixation with minimizing clumping of cells. Fixed the cells for 30 min at 4 °C. Cells were washed twice in PBS and centrifuged at a speed of 850 g. We resuspended the cells in 500 μl Propidium Iodide/Triton X-100 staining solution. (Freshly prepared 5 mL of 0.1% Triton X-100 (Sigma), 1 mg DNAse free RNase A (Sigma) and 0.2 mL of 50 μg/mL stock solution of Propidium Iodide (Roche). Incubated cells for 30 min at room temperature protected from light. Data were acquired using FACSCalibur.

### TOP-FLASH assay

We performed this procedure as described [10]. We plated T3M-4- shSEMA5A and -Control cells in 12-well dishes. The following day, we transiently transfected the cells with the TOP-flash or FOP-flash vector (0.5 μg/well) and a renilla vector as a transfection control (0.1 μg/well), *n* = 6 per cell line and vector. We obtained vectors from Promega (Promega, Madison, WI). Two days later, we assayed the cells for firefly/renilla luciferase using the Dual-Glo reagent (Promega), per the manufacturer’s instruction.

### Tumorigenic and metastasis assays

We purchased female athymic BALB/c nude mice (NCI-nu, 6–8 weeks old) from the National Cancer Institute. The Institutional Animal Care and Use Committee of the University of Nebraska Medical Center approved all the animal experiments performed in this. Cells were trypsinized and harvested for preparation of in vivo injection. We tested the viability of cells using trypan blue exclusion assay and used single cell suspensions of > 95% viability for injection.

Mice were injected subcutaneously into the lateral flank with 10^6^ cells suspended in 0.05 mL of HBSS for tumorigenic assay. We monitored tumor growth and euthanized animals when they were 10-weeks old. Tumors were measured with calipers twice a week. We calculated tumor volume (V) by taking a square of short diameter (W) multiplied by half of the long diameter (L) of the tumor (V = W^2^*L/2). Tumor tissues were harvested and processed for further analysis.

For studying tumor growth as well as metastasis, tumor cells were injected orthotopically into the pancreas of the mice. Mice were anesthetized, and we created a small incision for the injection of pancreatic tumor cells. We injected T3M-4 (5 X 10^5^ cells/0.05 mL HBSS) and CD18/HPAF (2.5 X 10^5^/0.05 mL HBSS) cells into the anterior lobe of the pancreas using a 1/2-cc U-100 insulin disposable syringe (EXEL International Inc., Los Angeles, CA). The appearance of a fluid bleb indicated a successful subcapsular intrapancreatic injection of tumor cells without leakage. The abdominal muscular layer was closed using 4–0 or 5–0 vicryl or polydioxanone (PDS), and the outer skin layer was closed with stainless steel wound clips. The animals tolerated the surgical procedure well, and no anesthesia-related deaths occurred. We removed wound clips at approximately 14 days post-surgical procedure. Mice were monitored for tumor growth and killed after 21 days post-injection. Primary tumors and metastases were resected and analyzed.

### Immunohistochemistry

We performed immunohistochemistry as described previously [11]. In brief, we incubated slides with αPCNA, (1:100; Santa Cruz, CA); or αSEMA5A [5] (1:50) primary antibodies in antibody diluent overnight at 4 °C. Next morning, we washed the slides and incubated with biotinylated anti-rabbit or anti-mouse secondary antibody (Vector Laboratories, Burlingame, CA) for 45 min. We quantitated the cell number for a particular stain by counting at least five different random fields in the same section at high resolution using a Nikon microscope.

### Statistical analysis

The significance of the data was determined by the Student’s *t*-test (two-tailed) for all in vitro studies. Comparisons between different mice groups were evaluated using Mann-Whitney U-test. The *p* < 0.05 was deemed significant. All statistical analyses were done using GraphPad Prism software (GraphPad Software, Inc., La Jolla, CA).

## Results

### SEMA5A knockdown induces a mesenchymal phenotype

**Fig. 1:**
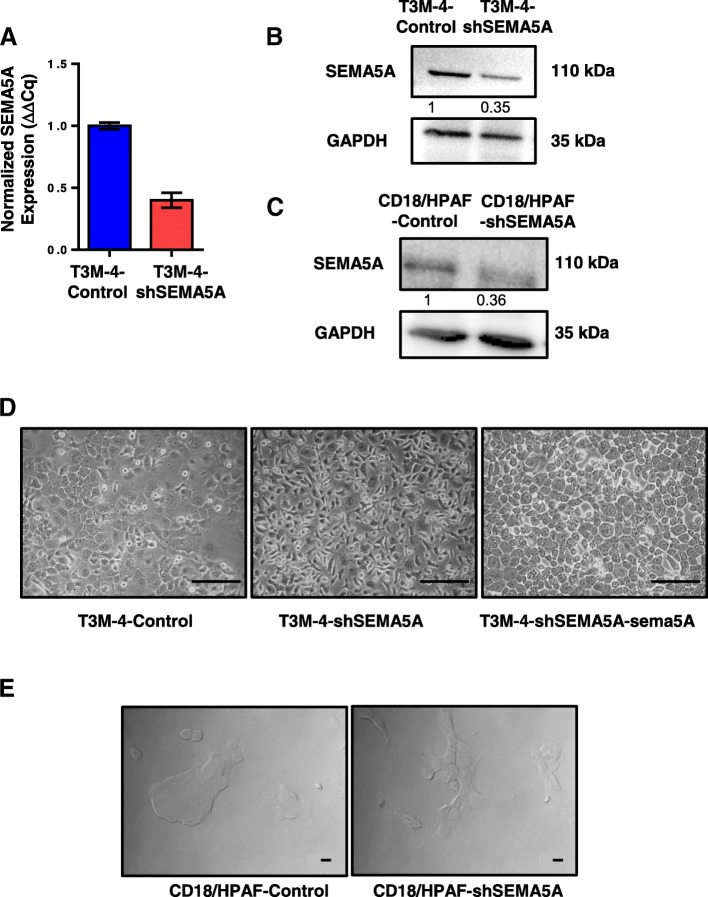
Confirmation of SEMA5A knockdown in PC cells and resulting mesenchymal morphology. **a**. Bar graph showing relative fold decrease in mRNA expression of *SEMA5A*. *HPRT* is used as a control by RT-PCR analysis. The values are mean relative fold changes ± Standard Error of Mean (SEM, bars). **b**-**c**. Western blot analysis of cell lysates of T3M-4-Control and T3M-4-shSEMA5A cells (**b**) and Control and CD18/HPAF-shSEMA5A cells (**c**) showing decreased SEMA5A protein expression with GAPDH as a loading control. Quantification of the protein of interest by the intensity of the bands with respect to their loading control was performed by Image J software. Bands were normalized on the T3M-4-Control cells or CD18/HPAF-Control cells. **d**-**e** Morphological changes upon SEMA5A knockdown. Cobblestone-like T3M-4-Control cells change to spindle-shaped cells in T3M-4-shSEMA5A cells. Furthermore, on expressing Flag-tagged mouse sema5A in T3M-4-shSEMA5A cells, the cells reverted back to their cobblestone appearance. Scale bar represents 100 μm in length (**d**). The transition of overlapping colonies of CD18/HPAF-Control cells to flat spindle-shape cells in CD18/HPAF-shSEMA5A cells. Scale bar represents 10 μm in length (**e**)

We knocked down SEMA5A expression in T3M-4 (Fig. 1a-b) and CD18/HPAF (Fig. 1c) cells using short hairpin (sh)RNA-mediated gene silencing. Reduced expression of *SEMA5A* at the RNA (Fig. 1a), as well as the protein levels in T3M-4-shSEMA5A (Fig. 1b) and CD18/HPAF-shSEMA5A cells (Fig. 1c) in comparison with their respective non-targeting Control, were observed. To our surprise, we found a marked difference in morphology between T3M-4-shSEMA5A and -Control cells. T3M-4-Control cells were epithelial and exhibited cobblestone-like appearance with closely opposed cell-cell junctions (Fig. 1d). In contrast, T3M-4-shSEMA5A cells showed relatively elongated morphology (Fig. 1d). We observed similar changes in morphology of CD18/HPAF cells (Fig. 1e) upon knockdown of SEMA5A. CD18/HPAF-Control cells formed tight, compact overlapping cellular colonies in comparison with their respective CD18/HPAF-shSEMA5A cells, which showed flat spindle-like structures (Fig. 1e).

### Loss of SEMA5A enhances cellular moti*lity*

**Fig. 2:**
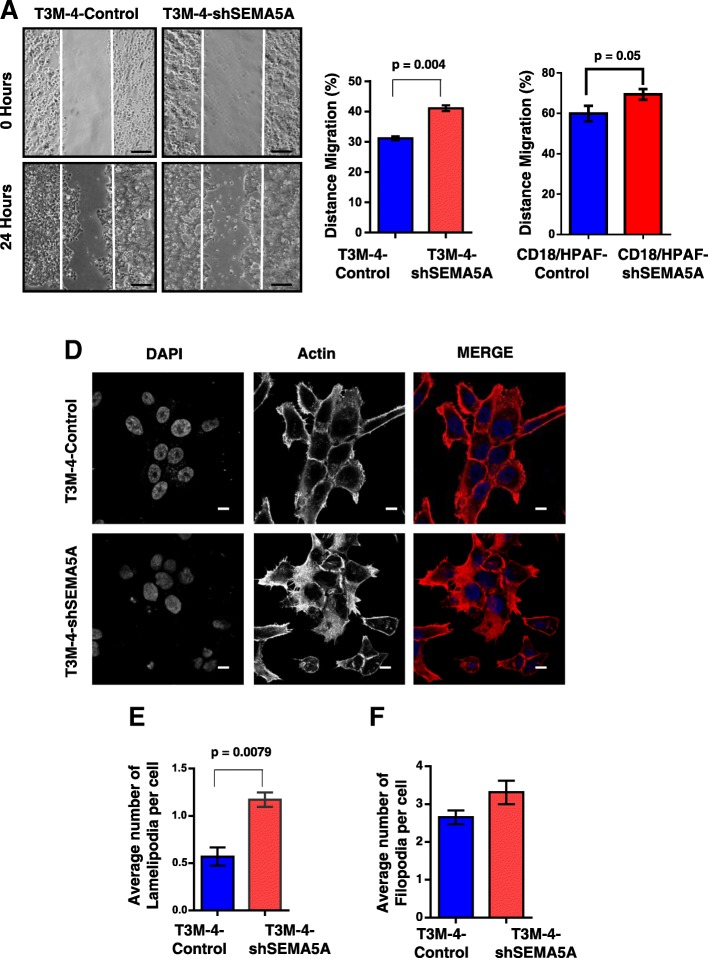
Loss of SEMA5A increases migration ability in PC cells. **a** Images are showing scratch assay of Control and T3M-4-shSEMA5A at 0 h and 24 h (Scale bar represents 100 μm in length). **b** Quantitation of scratch assay using ImageJ software showing a significant (*p* = 0.004) enhancement of migration in T3M-4 SEMA5A knock-down cells. **c** Quantitation of scratch assay showing a significant (*p* = 0.05) enhancement of migration in CD18/HPAF SEMA5A knock-down cells. The bars in the graphs represent the mean percentage of the distance of migration ± SEM and the significance of the data is calculated using Student’s t-test. **d** Images are showing staining of the actin cytoskeleton in T3M-4-Control and T3M-4-shSEMA5A cells with the increased number of lamellipodium- and filopodium- like structures in SEMA5A knockdown cells. Images were taken using an LSM 710 Zeiss Confocal Microscope. Scale bar represents 10 μm in length. **e** A bar graph is representing quantitation of an average number of lamellipodia in T3M-4-Control and T3M-4-shSEMA5A cells showing an increase in the number of lamellipodia formed (*p* = 0.0079) in SEMA5A knock-down cells. **f** Bar graph depicting the quantitation of filopodia, showing an increase in the number of filopodia in T3M-4 SEMA5A knock-down cells. The values in the graph represent a number of Lamellipodia formation/ or filopodia per peripheral cell and the error bars ± SEM. The significance of the data is calculated using the non-parametric Mann-Whitney U test

The gain of mesenchymal phenotype leads to enhanced migration and invasion ability of cells [12, 13]. We evaluated motility of the Controls and SEMA5A knockdown cells by scratch-wound assay (Fig. 2a-c, Additional file 2: Figure S1A). We observed that SEMA5A knockdown cells had increased motility in comparison with the Control in T3M-4 (*p* = 0.004, Fig. 2a-b) and CD18/HPAF cells (*p* = 0.05, Additional file 2: Figure S1A, Fig. 2c). We also observed increased migration/motility of T3M-4-SEMA5A in comparison with the T3M-4-Control using transwell migration assay (Additional file 2: Figure S1B). We also quantified lamellipodia and filopodia-like protrusions in the Control and T3M-4-shSEMA5A knockdown cells (Fig. 2d-f) and CD18/HPAF-Control and CD18/HPAF-shSEMA5A cells (Additional file 2: Figure S2C-D). We found a significantly higher number of lamellipodia (*p* = 0.0079, Fig. 2e) and an increase in the number of filopodia in T3M-4-shSEMA5A cells (Fig. 2f). Similarly, we also observed a higher number of lamellipodia (*p* = 0.001, Additional file 2: Figure S2D) in CD18/HPAF-shSEMA5A cells in comparison with the CD18/HPAF-Control cells. However, filopodia structures were difficult to quantify in CD18/HPAF cells. Also, we did not observe the qualitative difference in Fascin immunostaining in T3M-4-Control and T3M-4-shSEMA5A cells (Additional file 2: Figure S1E). The above data showing differences in morphology and motility ability of Controls and SEMA5A knockdown cells in T3M-4 and CD18/HPAF cells suggest that loss of SEMA5A in PC cells leads to loss of epithelial phenotype and a gain of mesenchymal characteristics. Also, we observed a significant increase in cell viability of T3M-4-shSEMA5A cells (Additional file 2: Figure S2A) and CD18/HPAF-shSEMA5A cells (Additional file 2: Figure S2B) in comparison with their respective Controls. In contrary, we observed a higher number of cells in G1/Go phase of cell cycle in CD18/HPAF-shSEMA5A cells in comparison with CD18/HPAF-Control cells (Additional file 2: Figure S2C-D).

### SEMA5A knockdown enhances metastasis

To evaluate the tumorigenic potential of SEMA5A knockdown cells, we first went ahead and injected SEMA5A knockdown and Control T3M-4 cells (10^6^) subcutaneously into the neck and flank regions of athymic nude, female mice (*n* = 6). Tumor growth was monitored twice weekly for three weeks. To our surprise, we did not observe a difference in tumor incidence (Additional file 2: Figure S3A), growth (Additional file 2: Figure S3B) or morphology (Additional file 2: Figure S3C) between SEMA5A knockdown and Control cells.

**Fig. 3:**
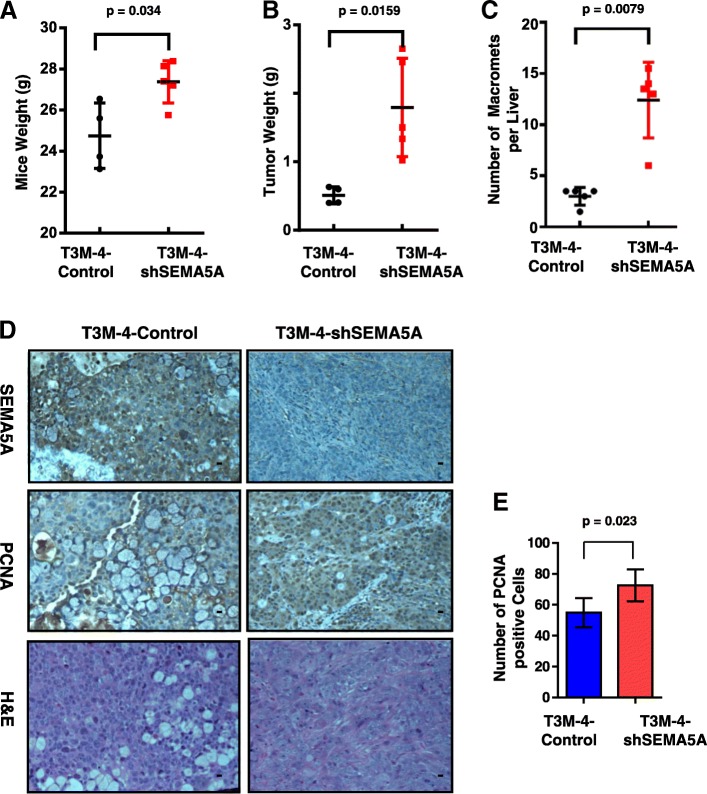
SEMA5A knockdown enhances tumor size and metastatic potential. **a**-**c** Graph showing increase in the average weight of the mice in grams (*p* = 0.034), (**a**) the average weight of primary tumor in grams (*p* = 0.0159) (**b**) and number of metastases per liver (p = 0.0079) **(c**) in mice orthotopically injected with T3M-4-shSEMA5A cells in comparison with mice injected with T3M-4-Control cells. The error bars in the graphs presented in this section represent ± Standard Deviation and significance of the data is calculated using Mann-Whitney U-Test. **d** Representative images showing SEMA5A and PCNA expression and H&E staining of orthotopic pancreatic tumors from T3M-4 shSEMA5A and Control cells. Scale bar represents 10 μm in length. Images are taken using a Nikon Eclipse E800 microscope. **e** Bar graph showing a higher number of proliferating tumor cells stained with PCNA (*p* = 0.023) in athymic mice orthotopically injected with T3M-4-shSEMA5A in comparison with those injected with Control cells. The error bars in the graphs represent ± SEM and * indicates statistical *p*-value less than 0.05 using Student t-test

Next, in addition to evaluation of tumorigenic potential, we also wanted to study the metastatic potential of SEMA5A knockdown and Control cells. With this objective, we orthotopically injected T3M-4-shSEMA5A and -Control cells and CD18/HPAF-shSEMA5A and -Control cells into athymic nude mice. We sacrificed the mice orthotopically injected with T3M-4-shSEMA5A and -Control cells in 21 days post-injection and examined tumor growth and metastasis. However, we sacrificed the mice orthotopically injected with CD18/HPAF-shSEMA5A and -Control cells in 35 days post-injection and examined tumor growth and metastasis. We observed a statistically significant increase in mice weight (*p* = 0.034) (Fig. 3a), tumor weight (*p* = 0.0159, Fig. 3b), and increase in the number of liver macrometastases (*p* = 0.0079, Fig. 3c) in mice injected with T3M-4-shSEMA5A than T3M-4-Control cells. In animals injected with CD18/HPAF cells, we observed no significant changes in mice weight (Additional file 2: Figure S4A) and tumor weight (Additional file 2: Figure S4B), but increase in the number of liver macrometastases (*p* = 0.0083, Additional file 2: Figure S4C) as well as liver micrometastasis (*p* = 0.0083, Additional file 2: Figure S4D) in mice injected with CD18/HPAF-shSEMA5A than CD18/HPAF-Control cells. Specifically, SEMA5A knockdown cells metastasized to the liver and spleen with a higher incidence in comparison with the Control cells in both T3M-4 (Additional file 2: Figure S4E) and CD18/HPAF cells (Additional file 2: Figure S4F). We also confirmed the maintenance of SEMA5A knockdown in the tumors formed by injection of T3M-4 cells by performing immunohistochemical staining of SEMA5A in tumor sections (Fig. 3d).

Lastly, we performed PCNA staining on Control and SEMA5A knockdown T3M-4 cells and observed higher PCNA positive cells (*p* = 0.023) in tumors of mice injected with T3M-4-shSEMA5A cells than those injected with T3M-4-Control cells (Fig. 3d, e).

### Loss of SEMA5A induces EMT markers and activates Wnt signaling

**Fig. 4:**
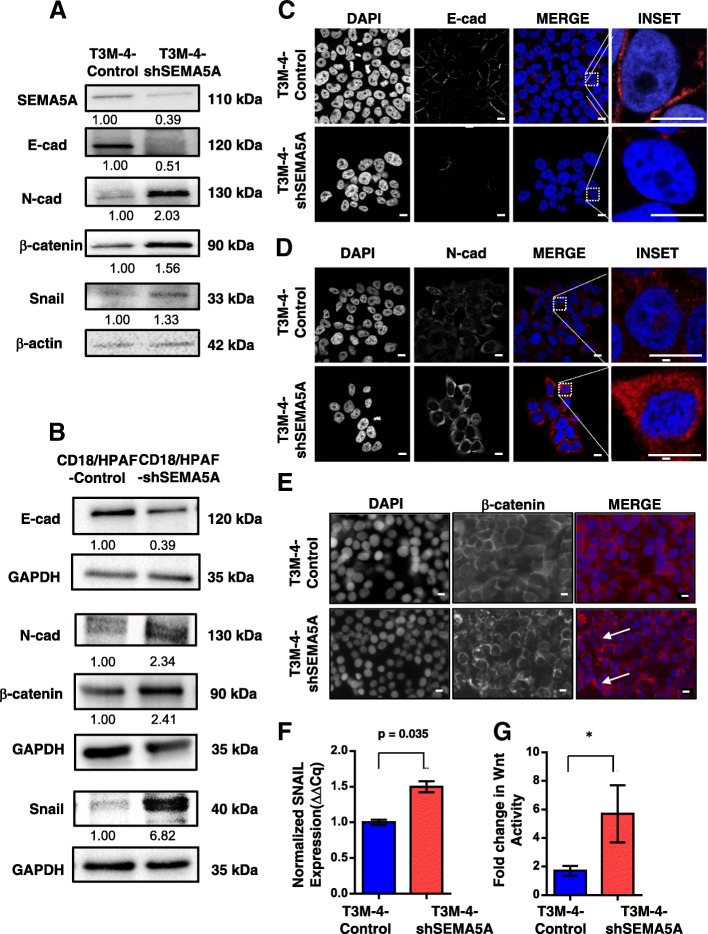
Loss of SEMA5A induces EMT and activates Wnt signaling in PC cells. **a-b** Western blot analysis of whole cell lysates of T3M-4-Control and -shSEMA5A cells (**a**) and CD18/HPAF-Control and –shSEMA5A cells (**b**). The analysis shows a decrease in protein level of SEMA5A, E-cad, increase in N-cad, Snail and β-catenin in T3M-4-shSEMA5A cells or CD18/HPAF-shSEMA5A cells in comparison with T3M-4-Control cells (**a**) or CD18/HPAF-Control cells (**b**) β-actin or GAPDH was used as a loading control. The intensity of the bands in western blot analysis was quantified by using Image J software and was normalized with respect to the T3M-4-Control cells or CD18/HPAF-Control cells. **c**-**e** Immunofluorescence analysis of E-cad (**c**), N-cad (**d**), and β-catenin (**e**) in T3M-4-Control and T3M-4-shSEMA5A cells. Immunofluorescence analysis is showing lower E-cad expression on the plasma membrane in T3M-4-shSEMA5A cells in comparison with Control cells (**c**). Immunofluorescence analysis of T3M-4-Control and T3M-4-shSEMA5A cells showing an increase in N-cad expression on the plasma membrane as well as cytoplasm in T3M-4-shSEMA5A cells (**d**). Images (**c-d**) were acquired using LSM 710 Zeiss Confocal Microscope. E-cad/N-cad is stained in red, and the nucleus is stained with DAPI (blue). **e** Immunofluorescence analysis is showing an increase in intensity and transition of β-catenin localization from the plasma membrane to the cytoplasm in T3M-4-shSEMA5A cells. β-catenin is localized to the plasma membrane in T3M-4-Control cells. Images are taken using a Nikon Eclipse E800 fluorescent microscope. β-catenin staining is depicted in red, and the nucleus is stained with DAPI in blue. Scale bar represents 10 μm in length. **f** Bar graph showing an increase in fold expression of transcription factor-SNAIL (*p* = 0.035) in T3M-4-shSEMA5A in comparison with Control cells evaluated using RT-PCR. The Ct values in RT-PCR were normalized to HPRT. Values are mean Fold changes ± SEM (bars) of two experiments. **g** Bar graph showing an increase in Wnt activity T3M-4-shSEMA5A than T3M-4-Control cells. TOP-FLASH was utilized, and this assay and values were normalized to both FOP and Renilla vector illustrating increased Wnt activity upon SEMA5A knockdown. The bars in the graphs represent fold changes ± Standard deviation and * - represents statistical p-value less than 0.05 using Student t-test

To characterize the observed morphological changes associated with the knockdown of SEMA5A at the molecular level, we first evaluated the expression of epithelial marker E-cad and observed decreased E-Cad expression in SEMA5A knockdown cells, in comparison with the Control cells (Fig. 4a-c). Similarly, the immunofluorescence analysis showed prominent plasma membrane staining of E-Cad in the T3M-4 and CD18/HPAF-Control cells, which was lower in T3M-4-shSEMA5A (Fig. 4c and CD18/HPAF-shSEMA5A cells (Additional file 2: Figure S5A).

Next, we evaluated the expression of mesenchymal markers in our Control and SEMA5A knockdown cells. We observed increased N-cad expression in the T3M-4-shSEMA5A cells (Fig. 4a) and CD18/HPAF-shSEMA5A cells (Fig. 4b) by Western blotting in comparison with their respective Control cells. We also observed more plasma membrane as well as cytosolic staining for N-cad in the T3M-4-shSEMA5A cells (Fig. 4d) in comparison with T3M-4-Control cells. Next, we evaluated expression levels of transcription factors Snail, which is a known suppressor of epithelial phenotype [14]. We observed higher levels of *SNAIL* mRNA transcripts (Fig. 4f, Additional file 2: Figure S5B) and protein (Fig. 4a, b) in SEMA5A cells knockdown cells in comparison with the Control cells.

Using immunofluorescence, we also observed an increased intensity and a shift of β-catenin localization from the plasma membrane to the cytoplasm (Fig. 4e, Additional file 2: Figure S5C) as well as increased expression of β-catenin by Western Blotting (Fig. 4a, b) upon SEMA5A knockdown. Furthermore, to confirm the presence of β-catenin in the nucleus, we went ahead and performed TOP-FLASH Wnt reporter assay. We observed a significant increase in Wnt activity in T3M-4-shSEMA5A cells (*p* < 0.05) in comparison with the Control cells (Fig. 4g).

### Knock-in of shRNA-resistant SEMA5A rescues the epithelial phenotype

**Fig. 5:**
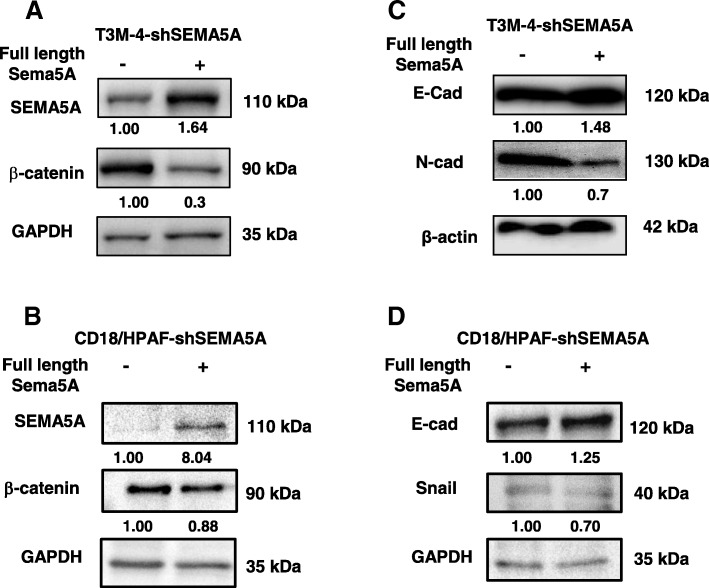
Overexpression of shRNA-resistant Sema5A rescues epithelial phenotype. **a**-**b** Western blot analysis is showing an increase in SEMA5A and decrease in β-catenin expression in T3M-4-shSEMA5A (**a**) and CD18/HPAF-shSEMA5A cells (**b**) transfected with control pBK-CMV vector and full-length mouse Sema5A. GAPDH was used as loading control. **c** Increase in E-Cad and decrease in N-cad protein expression with respect to β-actin as a loading control in T3M-4-shSEMA5A cells transfected control pBK-CMV vector and full-length mouse Sema5A. **d** Increase in E-Cad and decrease in Snail protein expression with respect to GAPDH as a loading control in CD18/HPAF-shSEMA5A cells transfected control pBK-CMV vector and full-length mouse Sema5A. The intensity of the bands in Western blot analysis was quantified using Image J software and was normalized with respect to the T3M-4-shSEMA5A or CD18/HPAF-shSEMA5A cells transfected with control pBK-CMV vector

To characterize the specificity of SEMA5A-induced mesenchymal characteristics, we performed experiments to re-establish SEMA5A expression in T3M-4-shSEMA5A and CD18/HPAF-shSEMA5A cells. Hence, we transiently over-expressed knockdown resistant full-length murine Sema5A construct in SEMA5A knockdown cells and observed a reversal of the gain of mesenchymal characteristics. Convincingly, SEMA5A re-establishment (Fig. 5a, b) rescued the decrease of E-Cad expression in SEMA5A knockdown cells (Fig. 5c, d). Similarly, the increase in expression of N-Cad (Fig. 5c), Snail (Fig. 5d), and enhanced expression of β-catenin (Fig. 5a, b) as seen in SEMA5A knockdown cells downregulated upon re-establishment of SEMA5A.

### Loss of SEMA5A activates PI3K/Akt signaling pathway

**Fig. 6:**
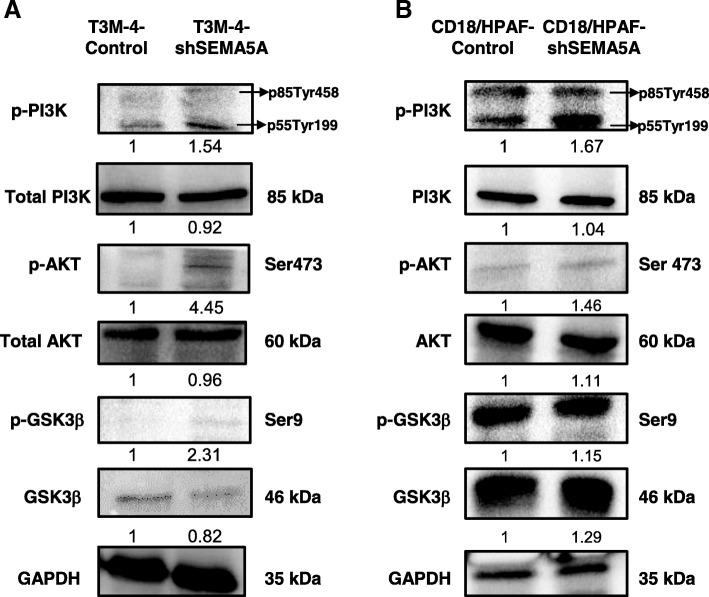
Loss of SEMA5A activates the PI3K/AKT pathway. **a**-**b** Western blots of whole cell lysates showing an increase in levels of p-PI3K (Ser 458), p-AKT (Ser 473) and GSK3β (Ser9) in T3M-4-shSEMA5A (**a**) in comparison with T3M-4-Control cells and CD18/HPAF-shSEMA5Acells (**b**) in comparison with CD18/HPAF-Control cells suggesting activation of PI3K/AKT signaling. GAPDH serves as a loading control. Western blots show no change in the total protein levels of PI3K, AKT, and GSK3β. The intensity of the bands in Western blot analysis was quantified by Image J using GAPDH as an internal control and was normalized with respect to the T3M-4-Control cells or CD18/HPAF-Control cells

We next examined the activation of PI3K/Akt signaling pathway (Additional file 2: Figure S6) as a possible mechanism of mesenchymal transition induced by knockdown of SEMA5A. We observed no changes in the protein levels of PI3-K and Akt levels between the Control and shSEMA5A of T3M-4 cells. However, p-PI3-K (p85-Tyr478 and p55-Tyr199) and p-AKT (Ser473) levels in T3M-4-shSEMA5A cells were found to be higher than their respective Control (Fig. 6a). Similarly, we observed higher phosphorylation of p-PI3-K (p85-Tyr478 and p55-Tyr199) and p-AKT (Ser473) levels in CD18/HPAF-shSEMA5A cells with slightly higher expression of PI3-K and AKT levels in CD18/HPAF-shSEMA5A cells (Fig. 6b). We also went ahead to evaluate the levels of total and p-GSK-3β (Ser9) in the Control and shSEMA5A of T3M-4 and CD18/HPAF cells. We observed higher p-GSK-3β in the T3M-4-shSEMA5A in comparison with T3M-4-Control cells (Fig. 6a) and higher total GSK-3β in CD18/HPAF-shSEMA5A cells than the Control cells (Fig. 6b).

## Discussion

Metastasis is one of the leading causes of mortality in PC [15]. Recent genomic characterization of pancreatic ductal adenocarcinoma revealed aberrations in axon guidance pathway genes [16]. Compelling evidence suggests that axon guidance molecules such as semaphorins are involved in cancer progression, invasion, and metastasis [3]. Previous reports from our laboratory have identified one such molecule SEMA5A, which demonstrated differential expression in pancreatic tumors in comparison with the normal pancreas [5, 8].

Furthermore, to understand the functionality of SEMA5A, exogenous expression of either full-length mouse membrane-bound Sema5A or an extracellular domain of mouse Sema5A (secretory) in Panc1 cell line resulted in enhanced metastasis of these cells suggesting a tumor-promoting role of SEMA5A in PC [5, 6]. Along with higher metastasis, Panc1 cells transfected with a secretory form of mouse Sema5A showed enhanced angiogenesis [6]. Also, Panc1 cells overexpressing a secretory form of mouse Sema5A secreted proangiogenic factors like CXCL8 and VEGF suggesting the involvement of SEMA5A in pathological angiogenesis [7].

The above evidence suggested a pro-tumorigenic role of SEMA5A in PC, and hence accordingly we hypothesized that downregulation of SEMA5A expression in PC cells would inhibit metastasis. Unexpectedly, we observed pronounced morphological changes associated with SEMA5A knockdown. Since metastatic progression requires a loss of contact with neighboring cells and the gain of both extracellular matrix invasion and migration properties; this process of morphogenetic reprogramming is termed “epithelial-mesenchymal transition” or EMT [17]. The observed changes in morphology upon SEMA5A knockdown suggested the induction of mesenchymal phenotype in T3M-4 and CD18/HPAF SEMA5A knockdown cells. For furthermore confirmation of our observed phenotype, we performed cell motility assays to test whether there are motility differences between Control and SEMA5A knockdown cells. By our expectation, we observed higher motility in SEMA5A knockdown cells in comparison with their respective Control cells. Also, the increase in migration ability is associated with changes in polarized assembly of the actin cytoskeleton resulting in the formation of protrusive and invasive structures (lamellipodia and filopodia) that help the cell to navigate through the extracellular matrix (ECM) and through/into the vasculature [18]. We also observed a significantly higher number of lamellipodia and increased number of filopodia in SEMA5A knockdown cells in comparison with the Control cells, thereby confirming the mesenchymal nature of SEMA5A knockdown cells. Given mesenchymal tumor cells are more tumorigenic, motile and hence more metastatic [12, 19], our experimental observations suggested that there is a possibility that loss of SEMA5A may promote metastasis. To address in which direction this balance will tip, we started with an evaluation of the tumorigenic potential of SEMA5A knockdown cells by subcutaneous injection into athymic nude mice. We did not observe a difference in tumor incidence and growth between SEMA5A knockdown and Control cells.

Next, we evaluated tumorigenic as well as the metastatic potential of SEMA5A knockdown cells by orthotopically injecting T3M-4- or CD18/HPAF-shSEMA5A and Control in nude mice. We observed enhanced metastatic potential of tumors formed by the injection of T3M-4-shSEMA5A and CD18/HPAF-shSEMA5A than those injected with T3M-4-Control and CD18/HPAF-Control cells. Additionally, we observed enhanced tumorigenic potential with a higher number of PCNA positive cells in sections of the tumor resulting from injection of T3M-4-sh-SEMA5A than those injected with T3M-4-Control cells. However, we failed to find any difference in tumor burden during subcutaneous injection of T3M-4-Control and T3M-4-shSEMA5A cells as well as orthotopic injection of CD18/HPAF-Control and CD18/HPAF-shSEMA5A cells. Thus, an increase in cellular migration ability with loss of SEMA5A was a more convincing reason for enhanced metastasis in both T3M-4 and CD18/HPAF cell-lines rather than differences in cell proliferation state. Also, enhanced metastasis points towards the possibility that loss of SEMA5A may have induced mesenchymal phenotype in PC cells. Before going ahead for characterization of this change in morphology, we confirmed that our injected T3M-4-shSEMA5A cells maintained SEMA5A knockdown by performing immunohistochemical staining of SEMA5A on tumor sections of mice injected with Control and knockdown cells. We observed lower SEMA5A expression in tumors resulting from injection of T3M-4-shSEMA5A cells in comparison with T3M-4-Control cells suggesting that our cells maintained the knockdown of SEMA5A.

Till now, we observed phenotypic and functional EMT changes associated with loss of SEMA5A molecule, however, the process of EMT leads to changes in molecular levels in a cell as well. The loss of the epithelial-like characteristics, includes loss of cell adherent junctional complexes, particularly the adherent junction protein E-cad, epithelial intermediate filament cytokeratin as well as cell polarization [15, 17, 18]. These changes follow the acquisition of mesenchymal-like characteristics, including a gain of the adherent junction protein N-cad, the cytoskeletal element vimentin, and upregulation of transcriptional factors such as zinc finger E-box binding homeobox 1, SNAlL, SLUG and TWIST [14, 17]. Thus, to validate our finding at the molecular level, we probed for changes in the expression of E-Cad (Epithelial marker), N-Cad and Snail (mesenchymal markers) protein by Western Blot and Immunofluorescence to test EMT. Both Western Blotting and Immunofluorescence revealed decreased E-Cad but increased expression of N-cad and Snail expression in SEMA5A knockdown cells, in comparison with Control cells suggesting that with the loss of SEMA5A, cells are undergoing EMT. Next, we probed for β-catenin as numerous studies have demonstrated that E-Cad forms a complex at the plasma membrane with β-catenin, which is essential for maintaining normal phenotype of epithelial cells [20–22]. Upon loss of E-Cad expression, β-catenin translocates from the plasma membrane to the cytoplasm where it can either undergo phosphorylation by GSK-3β and ultimately degraded or translocated into the nucleus. The relocalization of plasma membrane-bound β-catenin into the nucleus is both an indicator that epithelial cells are transitioning to the mesenchymal state and a central component in Wnt-responsive gene activation [22]. We observed a shift in β-catenin localization from the plasma membrane to the cytoplasm and nucleus upon SEMA5A knockdown and increased β-catenin protein expression in SEMA5A knockdown cells compared to the control.

Furthermore, we observed a significant increase in Wnt activity in SEMA5A knockdown cells using the TOP-FLASH Wnt reporter assay. These molecular changes suggested the induction of mesenchymal phenotype in T3M-4-shSEMA5A cells. Li et al. have published similar findings on SEMA5A in glioblastoma demonstrating that the treatment of glioblastoma cell lines with SEMA5A impaired cellular motility and promoted differentiation [23, 24]. In contrast with SEMA5A, other semaphorins like Semaphorin 7A in breast cancer cells [25] and Semaphorin 3E through PlexinD1 axis [26] in ovarian cancer cells are known to mediate EMT.

**Fig. 7:**
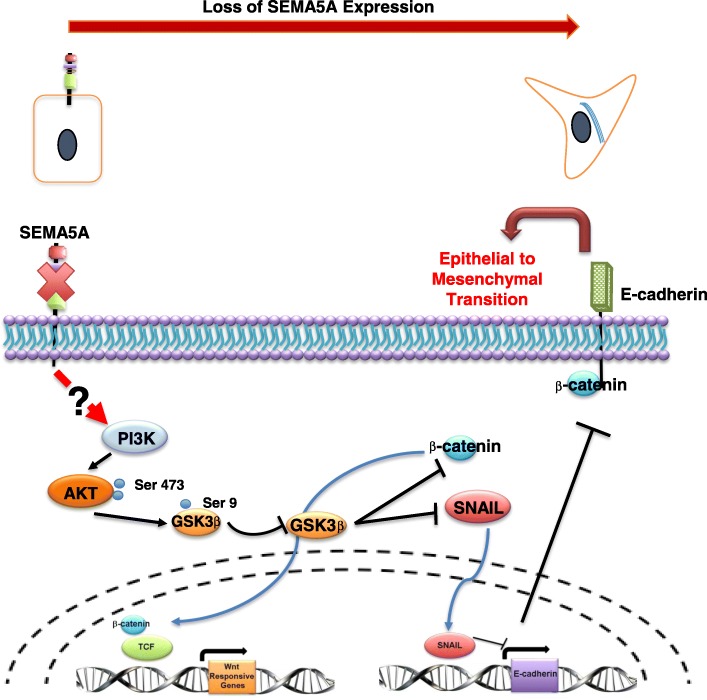
The putative mechanism is demonstrating how the loss of SEMA5A can trigger EMT. Representative schematic demonstrates the proposed mechanism of how the loss of SEMA5A leads to the activation of the PI3K/AKT pathway, which furthermore leads to inhibition of GSK-3β. This in turn results in the stabilization of β-catenin and Snail protein. Stabilization of β-catenin leads to active translocation of β-catenin from the cytoplasm into the nucleus, further leading to activation of WNT signaling. On the other hand, stabilization of Snail protein leads to the repression of transcription of E-cad. This suppression of E-cad transcription leads to a decrease in the E-cad protein levels, as well as leading to the induction of EMT

Lastly, we analyzed the loss of SEMA5A-induced EMT at the mechanistic level. We re-analyzed our results and observed stabilization or increase in expression of β-catenin and Snail protein in our knockdown cells. In the cytoplasm, phosphorylation of β-catenin and Snail occurs by GSK-3β, which target these molecules for degradation [27, 28]. Thus, one possible mechanism in our SEMA5A knockdown cells could be inhibition of GSK-3β, leading to an increase in β-catenin and Snail protein. With this rationale, we evaluated the inhibitory phosphorylation of GSK-3β at Ser9 and observed increased phosphorylation of GSK-3β at Ser9 in SEMA5A knockdown cells in comparison with the Control cells with no changes in the total protein level of GSK-3β. PI3K/AKT signaling is one of the signaling known to mediate inhibitory phosphorylation on GSK-3β [29]. With this knowledge, we chose to explore the PI3K/AKT pathway and observed increased phosphorylation of AKT at Ser473 and PI3K at Ser458, indicating activation of these proteins in SEMA5A knockdown cells in comparison with the Control cells. Again, there were no significant differences in the total protein levels of PI3K and AKT proteins in the Control and knockdown cells. However, our proposed mechanism (Fig. 7) needs further validation by utilizing inhibitors against PI3K and AKT molecules. Also, the possible link between SEMA5A and PI3K signaling requires investigation.

A possible explanation for the discrepancy that both gain of SEMA5A in primary tumor cells or loss of SEMA5A in metastatic PC cells result in higher metastasis may come from the involvement of two different pathways. To describe, in short, Type 1 TSP repeats, a unique structural feature of class 5 semaphorins, is known to activate the latent form of TGFβ, known player of EMT induction. A *Drosophila* homolog of SEMA5A has been shown to increase metastasis by activating the Dpp (TGFβ-like) pathway [30], suggesting increased SEMA5A will induce EMT. On the other hand in glioblastoma, loss of SEMA5A leads to Rac1 activation resulting in increased invasiveness, indicating that SEMA5A suppresses motility of cell [23, 24]. Furthermore, we have observed higher levels of SEMA5A in higher grade tumors and liver metastasis, however, SEMA5A expression was downregulated in lymph node metastasis, raising the possibility of the site and stage-dependent regulation of SEMA5A [8]. One of the limitations of the present study is the fact that we have used selected subpopulation of the cells with SEMA5A knockdown. Thus, to fully understand the complex role of functional SEMA5A in PC and metastasis, further experimentation using inducible expression system and better cellular model systems are needed.

## Conclusion

The present study taken together with our previous reports results suggest a bi-functional role for SEMA5A in PC progression and metastasis. Our previous observations indicate that in the primary tumor setting, SEMA5A promotes proliferation and angiogenesis, thereby enhancing metastasis. In the present finding, we observe that subsequent repression of SEMA5A induces EMT to facilitate motility, invasion, and metastasis. These results collectively suggest that SEMA5A might regulate the context-specific metastatic capacity of cancer cells.

## Additional files

Additional file 1: List of human primers used in the study. (DOCX 14 kb)

Additional file 2: **Figure S1.** Loss of SEMA5A increases migration ability in PC cells. A. Scratch assay showing higher cellular motility of CD18/HPAF-shSEMA5A cells. Scale: 100 μm. B. Transwell-migration assay showing higher cellular migration in T3M-4-shSEMA5A. C-D. Image (C) of Actin cytoskeleton and graph (D) showing an increased number of lamellipodium in CD18/HPAF-shSEMA5A cells. Scale: 10 μm. E. Immunofluorescence in T3M-4-Control and T3M-4-shSEMA5A cells showing no difference in Fascin localization. Scale: 10 μm. **Figure S2.** Loss of SEMA5A effects cellular viability and proliferation in PC cells. A-B. In vitro cell viability analysis of T3M-4- (A) and CD18/HPAF (B)-Control and -shSEMA5A cells showing higher viability of SEMA5A knockdown cells. C-D. Cell cycle analysis (C) showing a higher number of cells in S and G2/M phase (D) in CD18/HPAF-Control than CD18/HPAF-shSEMA5A cells. **Figure S3.** Subcutaneous injection of SEMA5A knockdown and Control T3M-4 cells. A-C Incidence of tumor-take (A), the growth kinetics (B) and presentation of Control and T3M-4-shSEMA5A (C). Scale: 10 μm. **Figure S4.** Orthotopic injections of T3M-4- and CD18/HPAF-Control and -shSEMA5A cells. A-C Graph showing no change in the average weight of the mice, (A) and the primary tumor (B) but, significantly higher number of macrometastases (C) and micrometastasis (D) in mice injected with CD18/HPAF-shSEMA5A. E-F. The incidence of tumor-take and metastasis in T3M-4- (E) and CD18/HPAF-shSEMA5A (F) and Control cells. **Figure S5.** Loss of SEMA5A induces EMT in PC cells. A. Immunofluorescence showing lower E-cad expression in CD18/HPAF-shSEMA5A. B. Graph showing an increase in fold expression of SNAIL in CD18/HPAF-shSEMA5A. C. Immunofluorescence showing loss of localization of β-catenin from plasma membrane and transition into the cytoplasm in CD18/HPAF-shSEMA5A cells. Scale bar: 10 μm. **Figure S6.** Representative schematic demonstrating that activation of PI3K/AKT pathway can lead to inhibition of GSK-3β resulting in stabilization of β-catenin and Snail. (PPTX 5406 kb)

## Abbreviations

DAPI: 4′, 6-diamidino-2-phenylindole, dihydrochloride
E-cad: E-cadherin
EMT: Epithelial-mesenchymal transition
H&E: Hematoxylin, and eosin
HPRT: Hypoxanthine-guanine phosphoribosyltransferase
N-cad: N-cadherin
PBS: Phosphate Buffer Saline
PC: Pancreatic cancer
PCNA: Proliferating cell nuclear antigen
SEMA5A: Human Semaphorin-5A
sema5A: Mouse Semaphorin-5A
shRNA: Short hairpin RNA
