# Supplementary material for: Semaphorin-5A maintains epithelial phenotype of malignant pancreatic cancer cells
Source: BMC Cancer. 2018 Dec 22;18:1283. doi: 10.1186/s12885-018-5204-x (PMC6303891; doi:10.1186/s12885-018-5204-x)
Supplement: Supplementary file 1 — List of human primers used in the study. (DOCX 14 kb) [file 12885_2018_5204_MOESM1_ESM.docx]

**Additional file 1**: **List of human primers used in the study:**

| **Gene** | **Orientation** | **Sequence (5’-3’)** | **Product size (bp)** |
| --- | --- | --- | --- |
| **SEMA5A** | Forward | GATCTATGGCATCTTTACCACCAA | 74 |
|  | Reverse | TGGCGTCAGGTTGAAGAC |  |
| **Snail1** | Forward | TTTACCTTCCAGCAGCCCTA | 207 |
|  | Reverse | CCCACTGTCCTCATCTGACA |  |
| **HPRT** | Forward | AGGGTGTTTATTCCTCATGGAC | 144 |
