# Supplementary material for: Semaphorin-5A maintains epithelial phenotype of malignant pancreatic cancer cells
Source: BMC Cancer. 2018 Dec 22;18:1283. doi: 10.1186/s12885-018-5204-x (PMC6303891; doi:10.1186/s12885-018-5204-x)
Supplement: Supplementary file 2 — Figure S1. Loss of SEMA5A increases migration ability in PC cells. A. Scratch assay showing higher cellular motility of CD18/HPAF-shSEMA5A cells. Scale: 100 μm. B. Transwell-migration assay showing higher cellular migration in T3M-4-shSEMA5A. C-D. Image (C) of Actin cytoskeleton and graph (D) showing an increased number of lamellipodium in CD18/HPAF-shSEMA5A cells. Scale: 10 μm. E. Immunofluorescence in T3M-4-Control and T3M-4-shSEMA5A cells showing no difference in Fascin localization. Scale: 10 μm. Figure S2. Loss of SEMA5A effects cellular viability and proliferation in PC cells. A-B. In vitro cell viability analysis of T3M-4- (A) and CD18/HPAF (B)-Control and -shSEMA5A cells showing higher viability of SEMA5A knockdown cells. C-D. Cell cycle analysis (C) showing a higher number of cells in S and G2/M phase (D) in CD18/HPAF-Control than CD18/HPAF-shSEMA5A cells. Figure S3. Subcutaneous injection of SEMA5A knockdown and Control T3M-4 cells. A-C Incidence of tumor-take (A), the growth kinetics (B) and presentation of Control and T3M-4-shSEMA5A (C). Scale: 10 μm. Figure S4. Orthotopic injections of T3M-4- and CD18/HPAF-Control and -shSEMA5A cells. A-C Graph showing no change in the average weight of the mice, (A) and the primary tumor (B) but, significantly higher number of macrometastases (C) and micrometastasis (D) in mice injected with CD18/HPAF-shSEMA5A. E-F. The incidence of tumor-take and metastasis in T3M-4- (E) and CD18/HPAF-shSEMA5A (F) and Control cells. Figure S5. Loss of SEMA5A induces EMT in PC cells. A. Immunofluorescence showing lower E-cad expression in CD18/HPAF-shSEMA5A. B. Graph showing an increase in fold expression of SNAIL in CD18/HPAF-shSEMA5A. C. Immunofluorescence showing loss of localization of β-catenin from plasma membrane and transition into the cytoplasm in CD18/HPAF-shSEMA5A cells. Scale bar: 10 μm. Figure S6. Representative schematic demonstrating that activation of PI3K/AKT pathway can lead to inhibition of GSK-3β resulting in stabil [file 12885_2018_5204_MOESM2_ESM.pptx]

## Slide 1
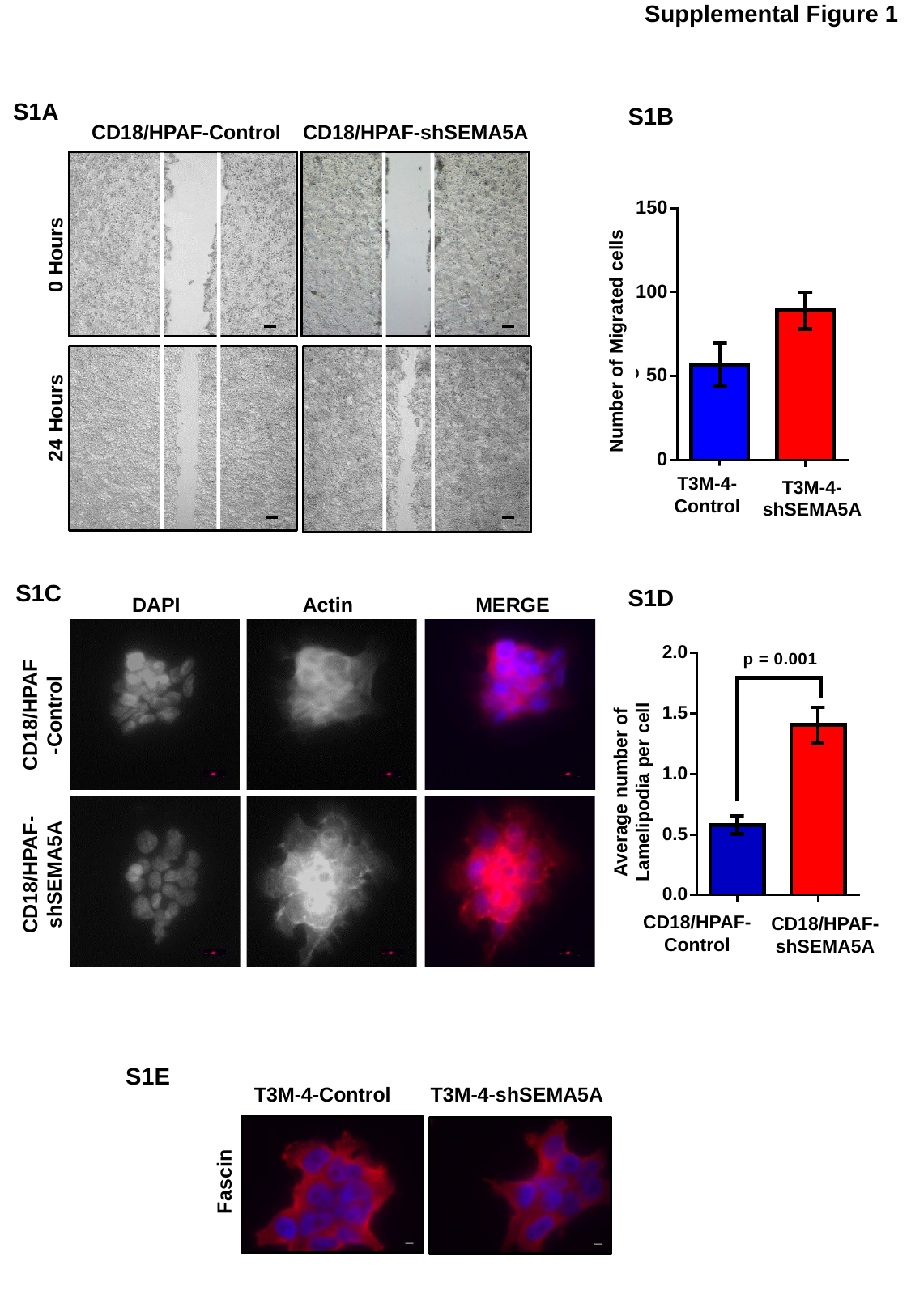

Supplemental Figure 1
CD18/HPAF-Control
CD18/HPAF-shSEMA5A
0 Hours
24 Hours
S1A
S1B
Number of Migrated cells
T3M-4-
Control
T3M-4-shSEMA5A
S1C
S1D
DAPI
Actin
MERGE
CD18/HPAF
-Control
CD18/HPAF-shSEMA5A
Average number of Lamelipodia per cell
CD18/HPAF-
Control
CD18/HPAF-shSEMA5A
S1E
T3M-4-Control
T3M-4-shSEMA5A
Fascin

## Slide 2
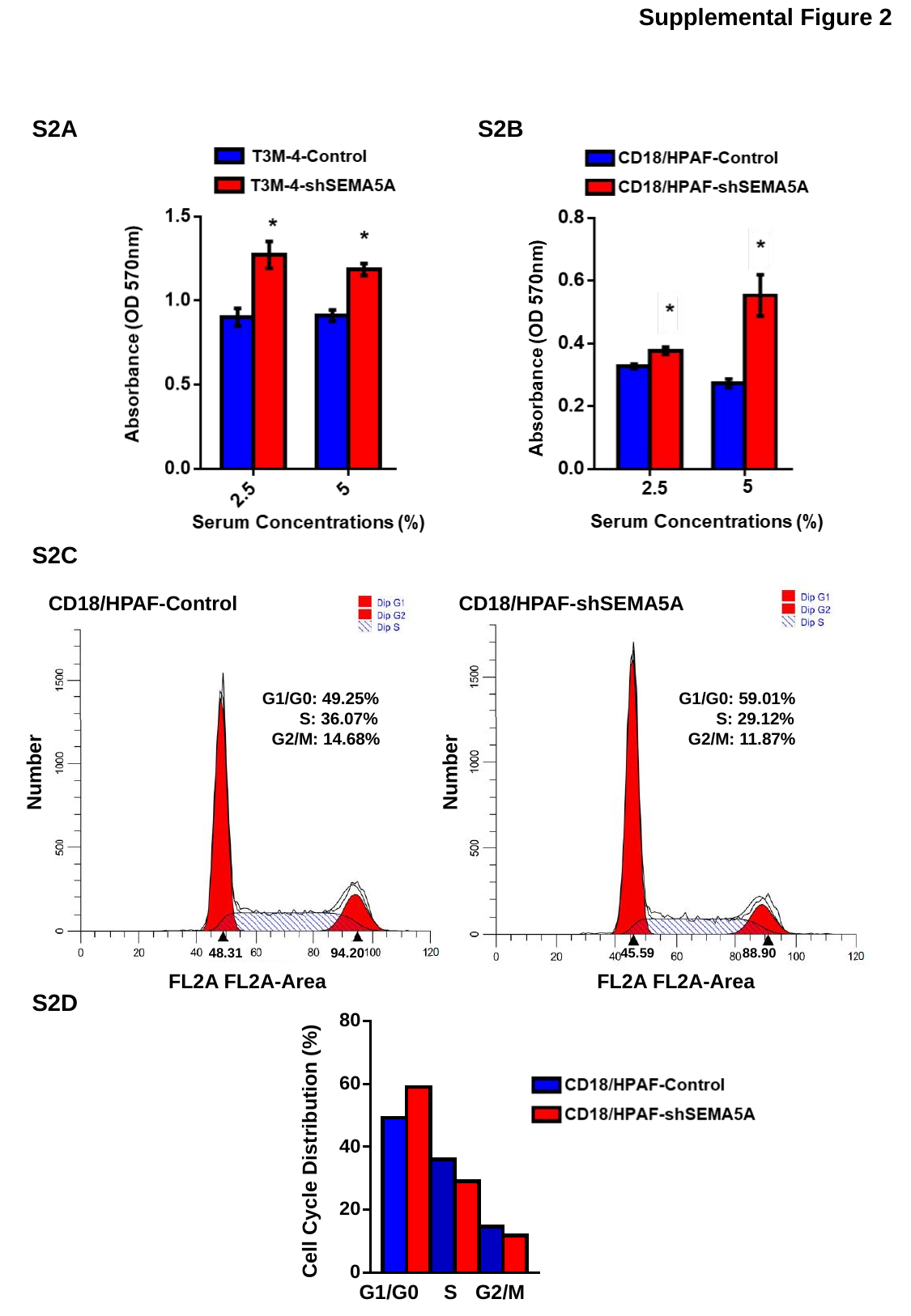

Supplemental Figure 2
S2A
S2B
S2C
CD18/HPAF-Control
CD18/HPAF-shSEMA5A
48.31
94.20
45.59
88.90
G1/G0: 49.25%
 S: 36.07%
 G2/M: 14.68%
G1/G0: 59.01%
 S: 29.12%
 G2/M: 11.87%
Number
Number
FL2A FL2A-Area
FL2A FL2A-Area
S2D
Cell Cycle Distribution (%)
G1/G0
S
G2/M

## Slide 3
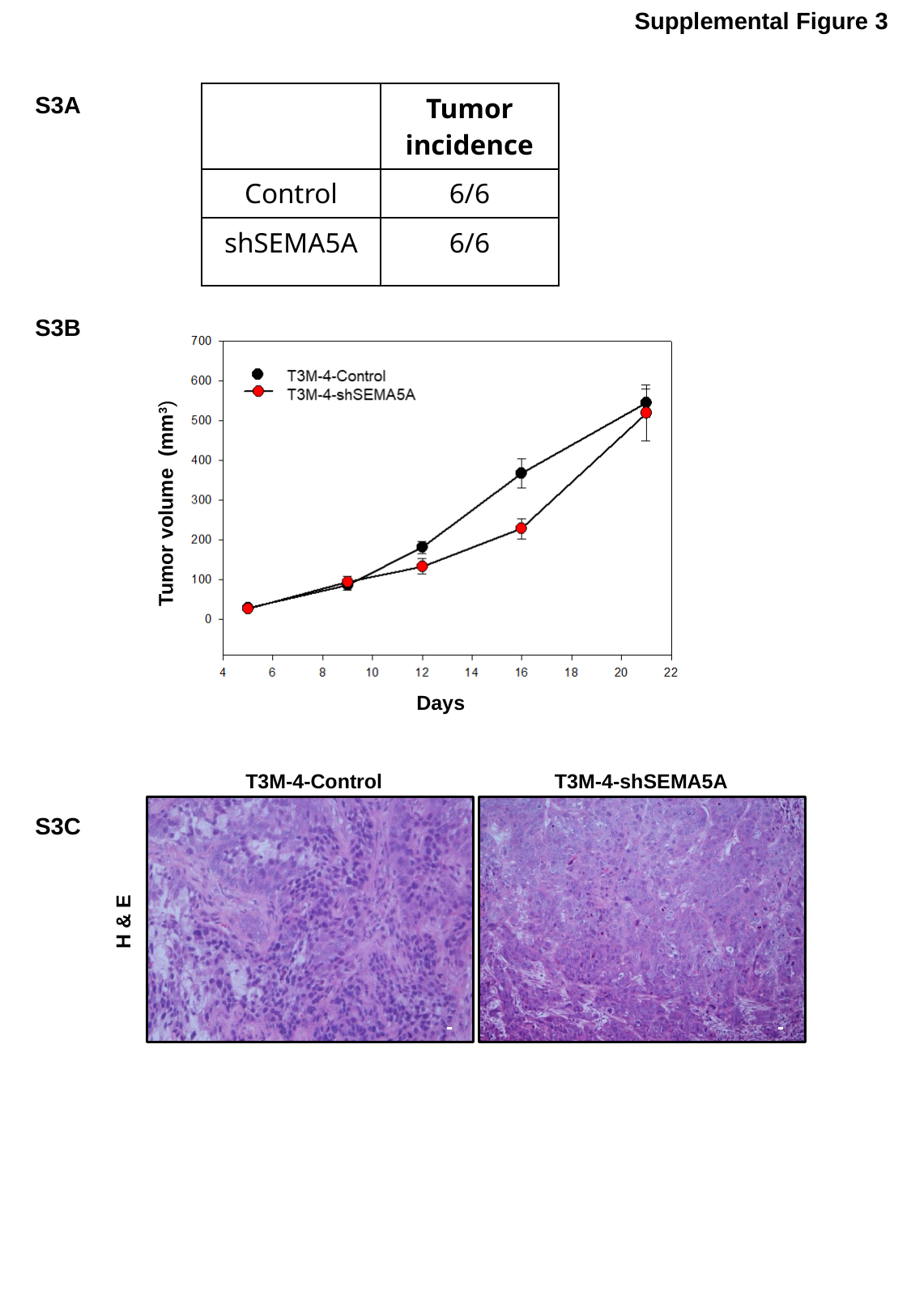

Supplemental Figure 3
| | Tumor incidence |
| --- | --- |
| Control | 6/6 |
| shSEMA5A | 6/6 |
S3A
Tumor volume (mm3)
Days
S3B
T3M-4-Control
T3M-4-shSEMA5A
S3C
H & E

## Slide 4
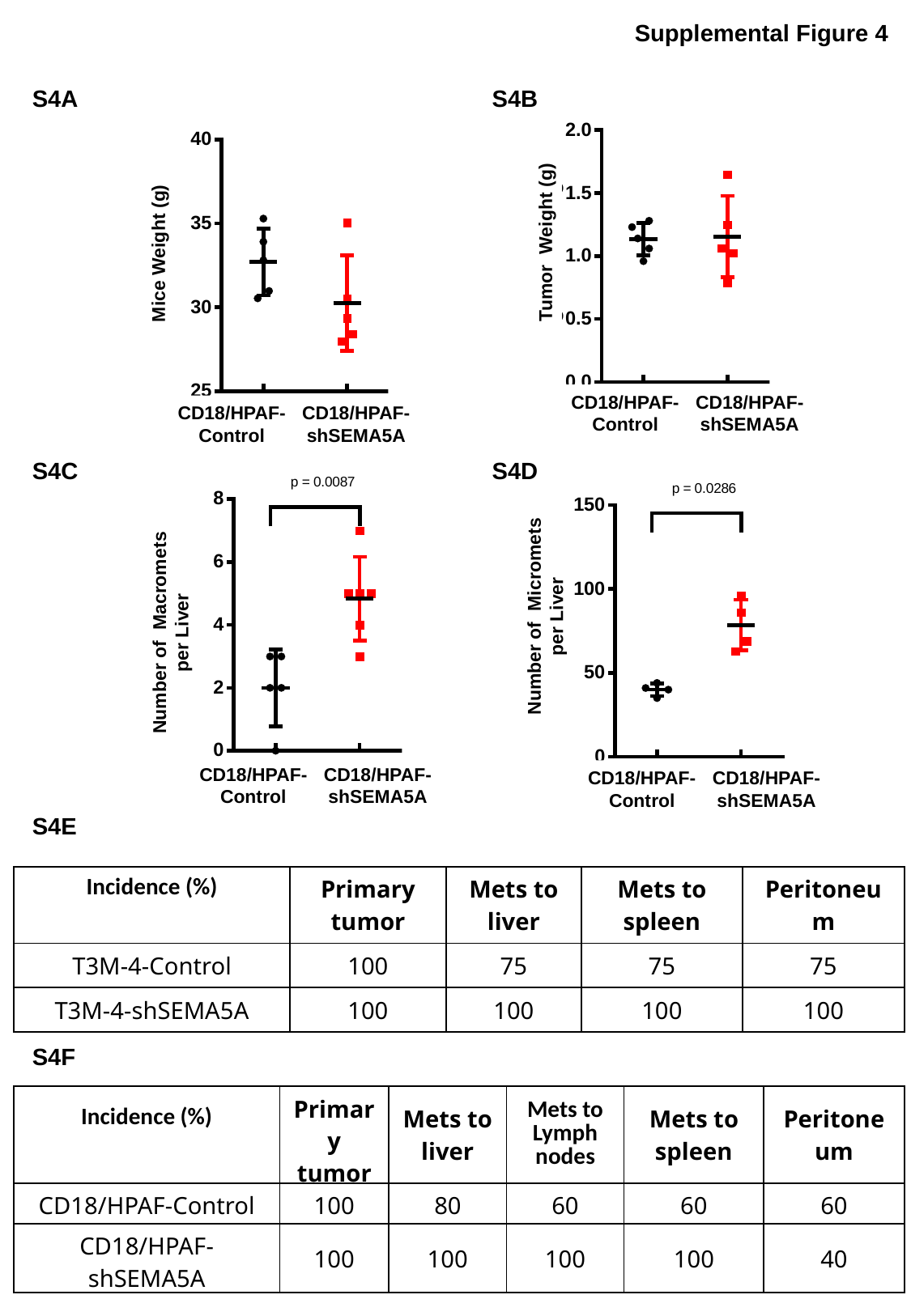

Supplemental Figure 4
S4A
S4B
 Mice Weight (g)
CD18/HPAF-
Control
CD18/HPAF-shSEMA5A
 Tumor Weight (g)
CD18/HPAF-
Control
CD18/HPAF-shSEMA5A
S4C
S4D
Number of Macromets per Liver
CD18/HPAF-
Control
CD18/HPAF-shSEMA5A
Number of Micromets per Liver
CD18/HPAF-
Control
CD18/HPAF-shSEMA5A
S4E
| Incidence (%) | Primary tumor | Mets to liver | Mets to spleen | Peritoneum |
| --- | --- | --- | --- | --- |
| T3M-4-Control | 100 | 75 | 75 | 75 |
| T3M-4-shSEMA5A | 100 | 100 | 100 | 100 |
S4F
| Incidence (%) | Primary tumor | Mets to liver | Mets to Lymph nodes | Mets to spleen | Peritoneum |
| --- | --- | --- | --- | --- | --- |
| CD18/HPAF-Control | 100 | 80 | 60 | 60 | 60 |
| CD18/HPAF-shSEMA5A | 100 | 100 | 100 | 100 | 40 |

## Slide 5
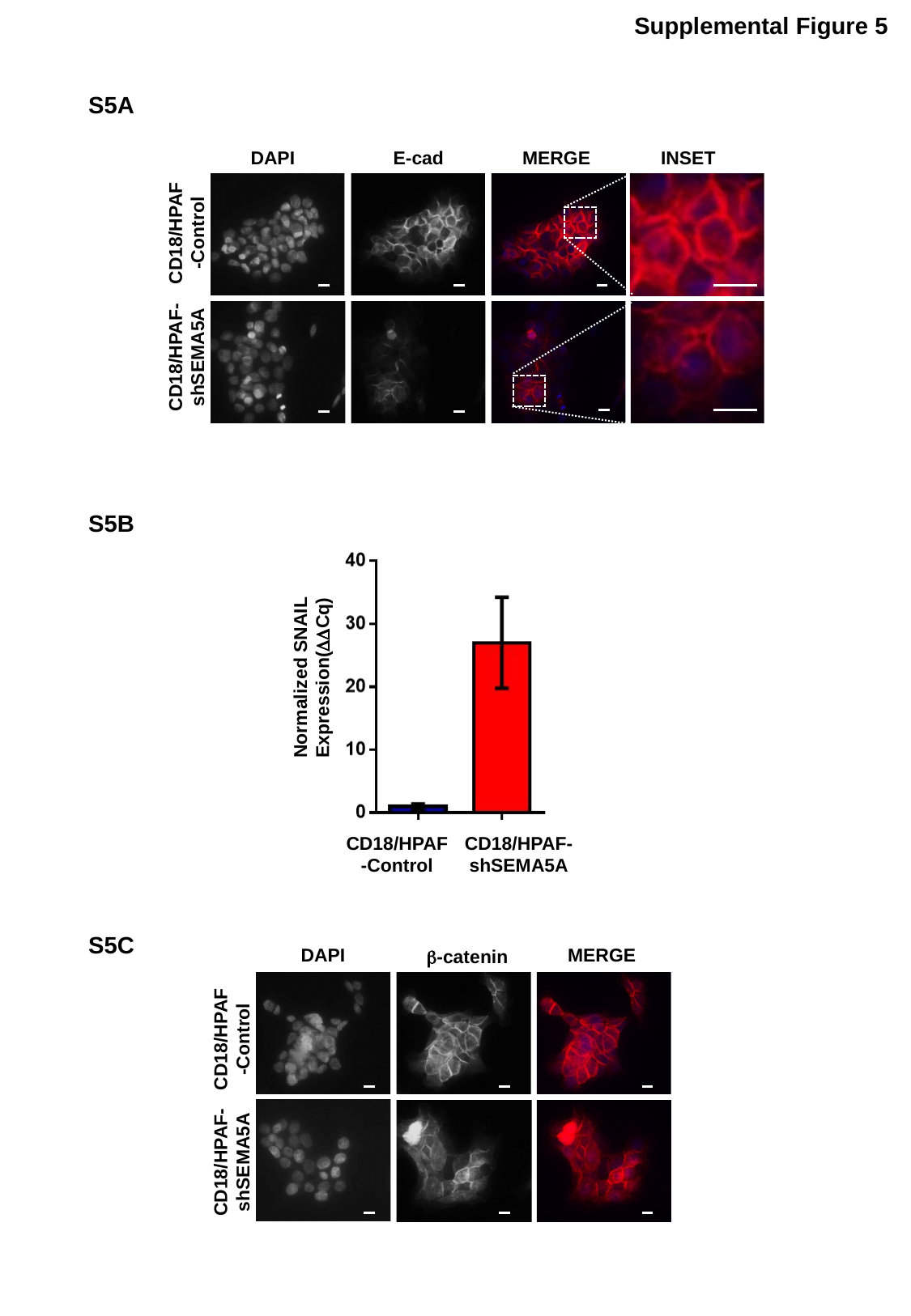

Supplemental Figure 5
S5A
DAPI
E-cad
MERGE
CD18/HPAF
-Control
CD18/HPAF-shSEMA5A
INSET
S5B
Normalized SNAIL Expression(DDCq)
CD18/HPAF
-Control
CD18/HPAF-shSEMA5A
S5C
DAPI
MERGE
b-catenin
CD18/HPAF
-Control
CD18/HPAF-shSEMA5A

## Slide 6
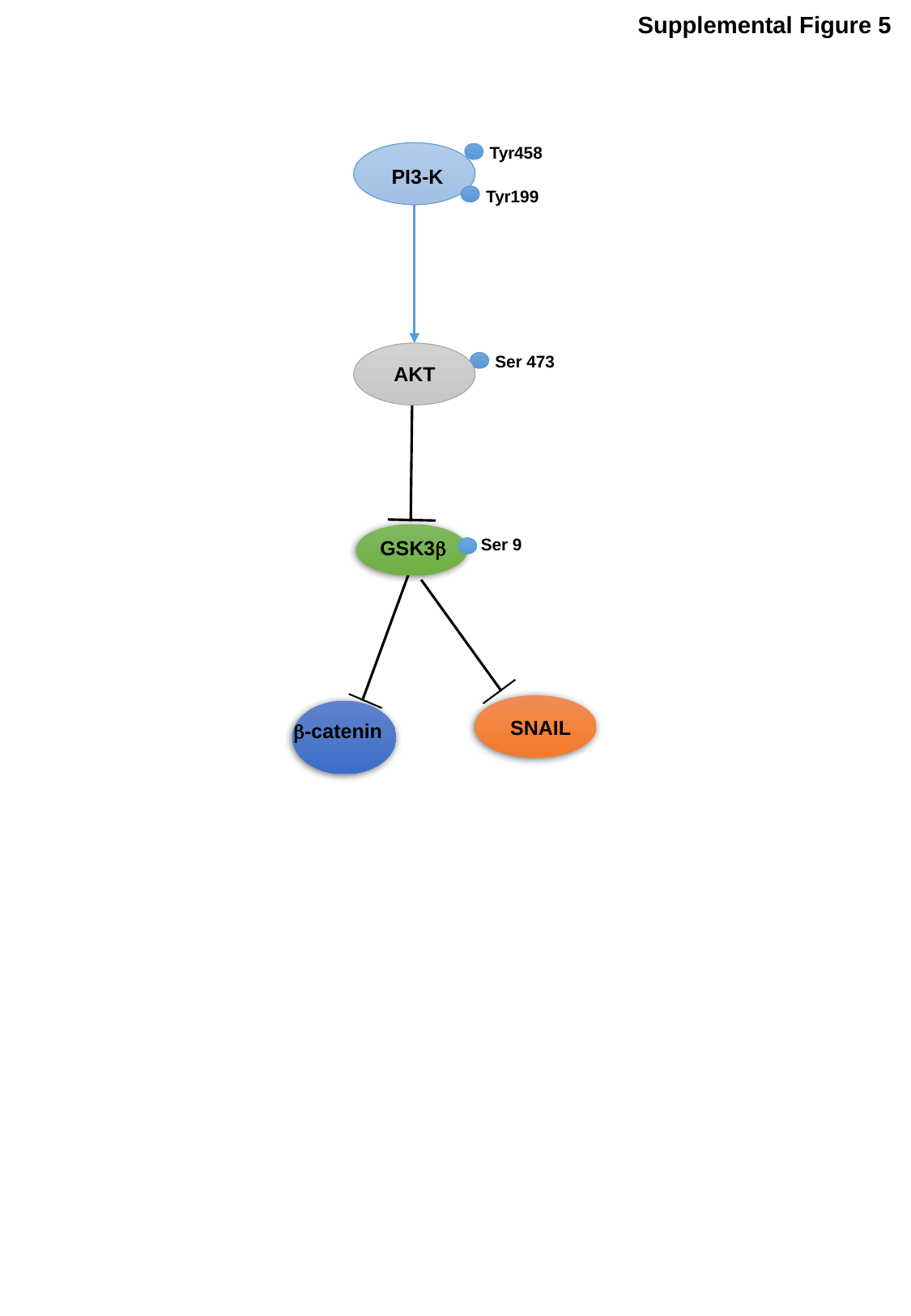

Supplemental Figure 5
Tyr458
PI3-K
Ser 473
AKT
Ser 9
SNAIL
b-catenin
GSK3b
Tyr199
